# Animal Chemistry; or Organic Chemistry in Its Applications to Physiology and Pathology

**Published:** 1842-10

**Authors:** 


### Art. XVI.

*Animal Chemistry ; or Organic Chemistry in its Applications to Physiology and Pathology.* By Justus Liebig, m.d. ph.d. f.r.s. m.r.i.a., Professor of Chemistry in the University of Giessen. Edited from the Author's Manuscript by William Gregory, m.d. f.r.s. m.r.i.a., Professor of Medicine and Chemistry in the University and King's College, Aberdeen.—*London,* 1842. 8vo, pp. 354.

Brief as is the period that has elapsed since the publication of this work, we believe that there are few scientific men to whom its name, at least, is not familiar; for few publications of modern date have so strongly excited public attention. We feel called on, therefore, to take the earliest opportunity of introducing our readers to its valuable contents; and this we shall do by presenting to them as full an analysis as our limits will allow, interspersed with a few critical remarks on some points in which we are disposed to think that the author has fallen into error; referring to the work itself all such as desire fuller information as to its details. If we do not unhesitatingly subscribe to all the commendations which have been bestowed upon his labours, it is because, after an attentive consideration of them, we feel assured that in some particulars they will require modification ; but, on the other hand, the same careful consideration has led us to attach very great importance to many views which have not struck others in the same light.

That Professor Liebig is *facile princeps* among the Organic Chemists of the day, we believe that no one will deny. To say that the present flourishing state of the science to which he has devoted himself is mainly due to his labours—that every subject he has touched upon has been elucidated by his inquiries—and that there is none, however obscure, upon which it may not be expected that he (and the fellow-labourers who are following in his path) will throw some light—is to give him but his due meed of praise. And to say, further, that the present work by no means disappoints the expectations we had formed of it, but in many respects goes beyond them, will be admitted, we think, as a sufficiently high expression of our opinion of it. We regard its value as rather consisting in the novelty of the path of inquiry which it opens up, and in the numerous suggestions which arise as branches of that path, than in the results actually obtained. This is the author's own appreciation of his labours; and we regard it as a merit rather than as a fault, that he has thus early made them public, since he thereby enlists as his assistants in the prosecution of his inquiries all those who are at present devoting themselves to investigations of a more desultory character.

The chief fault which we have to find with the work is, that the author does not confine himself sufficiently within the limits of chemistry—the subject of which he is confessedly the greatest master—but connects his chemical statements with speculations in physiology and pathology—in which, as it seems to us, he generally fails. He appears to have done this somewhat unwittingly; for the following remark, which occurs in the dedication to the British Association, seems to show that he is well aware of his true province:

"When the chemist shows, for example, that the elements of bile, added to those of the urate of ammonia, correspond exactly to those of the blood, he presents to us a fact which is independent of all hypothesis. It remains for the physiologist to determine, by experiment, whether the conclusions drawn by the chemist from such a fact be accurate or erroneous. And whether this question be answered in the affirmative or in the negative, the fact remains, and will some day find its true explanation."

Our author is evidently possessed with the idea, that by chemistry alone can physiology be now advanced. It is certainly well for science that its labourers should restrict themselves to particular departments: and it is pleasant to meet with enthusiasts, who, by their exclusive devotion to one set of ideas, are pretty sure to work out all that is valuable in them. But we regret to see a man who occupies a position so eminent in the scientific world as that on which Liebig stands, committing himself to the following extraordinary statements, which imply a degree of exclusiveness that we should not have expected from him:

"During the last five-and-twenty years physiology has acquired new ways and methods of investigation within her own province; and it is only *the exhaustion of these sources of discovery* which has enabled us to look forward to a change in the direction of the labours of physiologists. The time for such a change is now at hand; and a perseverance in the methods lately followed in physiology would now, from the want which must soon be felt of fresh points of departure for researches, render physiology more extensive, but neither more profound or more solid.. .............. The study of the uses and functions of the different organs, and of their mutual connexion in the animal body, was formerly the chief object of physiological researches; but lately this study has fallen into the back ground. These researches have yielded us the most valuable results, in relation to the recognition of the dissimilar forms and conditions to be found in the healthy and in the diseased organism; but they have yielded no conclusions calculated to give us a more profound insight into the essence of the vital phenomena." (Preface, pp. 11-12.)

Now we fearlessly assert that at no time has the advance of pure physiology (from which we exclude chemistry) been so rapid, as during the last five-and-twenty years; and that at no part of that period has this rapidity been so extraordinary as during the last five years. So that, so far from apprehending an exhaustion of our methods of research, we ourselves anticipate a continually-increasing harvest of new results. The true relation of chemistry to physiology appears to us to be entirely misunderstood by Liebig; and we shall endeavour to explain it in a few words. Chemistry may fairly take cognizance of all those processes, by which the vegetative functions of the living organism are brought into relation with the world around. It may, on the one hand, trace the conversion of the alimentary materials into organizable products; but there it ends: it can throw no light upon the process of organization, or upon the new (vital) properties which are called forth by it. On the other hand, it can take up the same materials, in the act of being restored to the inorganic world by the metamorphosis of the tissues; and it may give an account of all the changes which these materials undergo, up to the time when they are finally cast off by some of the excretory processes. But of the *vital* functions of the organized tissues, which it is the peculiar province of the physiologist to investigate, chemistry can give no account whatever; and these constitute a field which may well find room for the united labours of any number of investigators.

In thus limiting the domain of chemistry, however, we shall not be found to underrate its practical importance; for we leave to it the investigation of the whole range of effects produced on the living organism by external agents, whether alimentary materials, morbific causes, or remedial substances; and it will be found that all the results obtained by Liebig have reference to one or other of these departments.

We shall not follow, in our analysis, exactly the order of the work, because we should thereby be led into much repetition. It might be wished that the editor had reduced it to a form better adapted for being readily comprehended by the student. We do not regard it as by any means a readable book. The arrangement seems to us to be far from good. The train of thought is often interrupted by digressions; and it is frequently difficult to keep a clear view of the main argument. This must be our excuse, if we should prove to have sometimes misunderstood our author.

On many most important questions we have not ventured to offer an opinion, because they must be regarded as still *sub judice*, and as to be decided rather by chemical than by physiological evidence. And we may here especially advert to one of our author's fundamental assumptions—that of the absence of power in the animal body to convert the non-azotized elements of food into azotized compounds—as requiring confirmation. It is well known that the vegetable, by a supply of ammonia, can form gluten out of what would otherwise have been deposited as starch; and it is not enough to assert dogmatically that the animal is destitute of this power. It has been distinctly stated by Dr. Prout, that he has found albumen in the duodenum when none was formed in the stomach; and this circumstance has led him to the conclusion that a highly-azotized substance may be secreted into the duodenum, for the purpose of being united with the non-azotized constituents of the food, to form a compound adapted for the nutrition of the tissues. If this assertion be substantiated, a large part of Liebig's doctrine of nutrition and secretion will fall to the ground.

The discussion of the details of these functions is appropriately introduced by a few remarks on the vital force, to which a large proportion of the phenomena exhibited by living beings must be attributed; our author's views as to the nature of this force are more clearly set forth, however, in a subsequent part of the work, from which we derive the following account of them.

1. *Vital force.* The various changes exhibited in the growth of a plant or animal—involving as they do an immense variety of individual actions, no one of which can be entirely explained by any application of the known laws of physics or chemistry, whilst many of them take place in direct opposition to these laws—cannot be philosophically viewed in any other light, than as dependent on a cause or set of causes peculiar to themselves; but having strong analogies with the agents which operate on inorganic matter. "If the vital phenomena be considered as manifestations of a peculiar force, then the effects of this force must be regulated by certain laws, which laws may be investigated" like those of physics or chemistry. It manifests itself in the growth of the organism, and in its resistance to those external agencies which would tend to disintegrate the mass. It further presents itself as a cause of motion, and of change in the form and structure of the material substances which serve as food, disturbing and abolishing the chemical equilibrium in which they previously were, and causing their elements to arrange themselves in new forms, so as to produce new compounds. Of these compounds, some are identical in composition with the living tissue, and are made to become a part of that tissue by the attractive power of the vital force; whilst others, whose composition differs from that of the living tissue, are removed from the situation in which they are found, and are passed off as excretions by other parts of the body.

"The manifestations of the vital force are dependent on a certain form of the tissue in which it resides, as well as on a fixed composition in the substance of the living tissue. The capacity of growth in a living tissue is determined by the immediate contact with matters adapted to a certain decomposition, or the elements of which are capable of becoming component parts of the tissue in which vitality resides. The phenomenon of growth or increase in the mass presupposes that the acting vital force is more powerful than the resistance which the chemical force opposes to the decomposition or transformation of the elements of the food." (p. 198.)

Our author then goes on to point out that, in order to attain a clear conception of the manifestations of the vital force, differing as they do so remarkably amongst themselves, it is necessary to bear in mind that every known force is recognised by two conditions of activity, which present different phenomena to the attention of the observer. Force exerts itself in matter either in the form of resistance to external causes of motion or of change in form and structure, or as a moving force when no resistance is opposed to it or when that resistance is overcome by it. In the former case there is a state of equilibrium; and we only know of the existence of these forces by the result of the disturbance of the equilibrium through any external agency. In the latter case we recognise the force by its obvious effects.

"Both these manifestations of activity may be observed in that force which gives to the living tissues their peculiar properties. The vital force appears as a moving force or cause of motion, when it overcomes the chemical forces (cohesion and affinity) which act between the constituents of food, and when it changes the position or place in which their elements occur; it is manifested as a cause of motion in overcoming the chemical attraction of the constituents of food, and is, further, the cause which compels them to combine in a new arrangement, and to assume new forms." (p. 204.)

Its existence in the passive condition is evinced by the power of resisting those forces, which would otherwise disintegrate the structure.

We are not left to suppose, however, that this force has any existence distinct from the matter on which it acts. The *analogy* between vital force and chemical force is extremely strong; although every idea of their *identity* is contravened by the fact of their continually-opposing action. No philosopher has ever proclaimed the existence of a chemical force or principle as an entity separate from matter, and controlling its operations *ab externo;* since all are agreed that it is nothing more than a result of certain properties of matter, which are involved in our fundamental conception of it. Equally unphilosophical would it be to endeavour to abstract a vital force or principle from those peculiar forms of matter on which it operates. It is most justly urged by Liebig that—

"As the manifestations of chemical forces seem to depend on a certain order in which the elementary particles are united together, so experience tells us that the vital phenomena are inseparable from matter; that the manifestations of the vital force in a living part are determined by a certain form of that part and by a certain arrangement of its elementary particles. If we destroy the form or alter the composition of the organ, all manifestations of vitality disappear. *There is nothing to prevent us from considering the vital force as a peculiar property, which is possessed by certain material bodies, and becomes sensible when their elementary particles are combined in a certain arrangement or form.* This supposition takes from the vital phenomena nothing of their wonderful peculiarity; it may therefore be considered as a resting-point, from which an investigation into these phenomena and the laws which regulate them may be commenced, exactly as we consider the properties and laws of light to be dependent on a certain luminiferous matter or ether, which has no further connexion with the laws ascertained by investigation.............A living part acquires, on the above supposition, the capacity of offering and overcoming resistance, by the combination of its elementary particles in a certain form; and as long as its form and composition are not destroyed by opposing forces, it must retain its energy uninterrupted and unimpaired. When by the act of manifestation of this energy in a living part, the elements of food are made to unite in the same form and structure as the living organ possesses, then these elements acquire the same powers. By this combination the vital force inherent in them is enabled to manifest itself freely, and may be applied in the same way as that of the previously existing tissue." (pp. 209-10.)

The views here upheld are *identically* the same with those which were some years since maintained by Dr. Carpenter, in his essay "On the Laws regulating Vital and Physical Phenomena," and since embodied in his Principles of Physiology. The doctrine contained in the sentence in italics is that which it was the whole object of the essay to establish; and even the verbal correspondence is extremely striking, as the following quotation will show:

"But there is nothing inconsistent with our knowlege of the physical properties of matter, in the belief that all matter (or each at least of those forms of it capable of being assimilated) is also endowed with vital properties; which remained undisplayed or occult, until brought into action by being subjected to those conditions which a living organized system can alone afford." (Edinb. New Phil. Journal, April, 1838.)

Whatever merit, therefore, is due to the idea, (which, if correct, is indisputably an important step in generalization,) it may be fairly claimed by Dr. Carpenter; but we feel sure that, far from attempting to fix upon Liebig a charge of literary plagiarism, he will rejoice to find his position strengthened by the independent testimony of so eminent a philosopher. A more valuable example could scarcely be adduced of the fact, that fellow-labourers in the pursuit of truth, engaged in the same inquiries, and with the same spirit, are likely to arrive at the same conclusions, and may express them in language almost identical.

II. *The phenomena of motion in the animal organism.* Some speculations upon this subject are connected by Liebig with his propositions regarding the vital force; and though, as we conceive, they are weakened in their application by his neglect of certain fundamental physiological truths, yet they are of so interesting a character that we cannot pass them over in silence. The guesses of a Liebig, even when proved to be partly erroneous, are more worthy of attention than the pretending dogmata of shallower minds; and we shall find that these contain many ideas of high value, which, when more correctly applied, may be found to afford the true expression of the causes of the phenomena in question. The following passage is continuous with that last quoted:

"If now we bear in mind that all matters which serve as food to living organisms are compounds of two or more elements, which are kept together by certain chemical forces—if we reflect that, in the act of manifestation of force in a living tissue, the elements of the food are made to combine in a new order—it is quite certain that the momentum of force or of motion in the vital force was more powerful than the chemical attraction existing between the elements of the food. The chemical force which kept the elements together acted as a resistance, which was overcome by the active vital force. Had both forces been equal, no kind of sensible effect would have ensued. Had the chemical force been the stronger, the living part would have undergone a change. If we now suppose that a certain amount of vital force must have been expended in bringing to an equilibrium the chemical force, there must still remain an excess of force by which the decomposition was effected. This excess constitutes the momentum of force in the living part, by means of which the change was produced; by means of this excess the part acquires a permanent power of causing further decompositions, and of retaining its condition, form, and structure, in opposition to external agencies. We may imagine this excess to be removed, and employed in some other form. This would not of itself endanger the existence of the living part, because the opposing forces would be left in equilibrio; but by the removal of the excess of force the part would lose its capacity of growth, its power of causing further decompositions, and its ability to resist external causes of change. If, in this state of equilibrium, oxygen (a chemical agent) should be brought in contact with it, then there would be no resistance to the tendency of the oxygen to combine with some element of the living part, because its power of resistance has been taken away by some other application of its excess of vital force." (p. 212.)

In plants there is no demand upon the vital force for the maintenance of the form and structure of their non-azotized constituents; since these, when once formed, have little or no tendency to disintegration, except when affected by external agents. Their azotized principles, however, exhibit the same tendency to spontaneous decomposition as do those of animals, and need, therefore, in Liebig's opinion, a constant exertion of the vital force for their preservation. During every period of the life of a plant, the available vital force (that which is not neutralized by resistance,) is expended only in one form of vital manifestation—that of growth or increase of mass, or the overcoming of resistance; and it is interesting to observe, that those species which have the longest life and the greatest capacity for extension, are those which form the least amount of azotized principles, whilst those that produce the greatest quantity of these (as the cerealia and the fungi) have but a brief individual duration. But in animals, besides this source of expenditure, there is another, of still greater importance—the production of motion, whether this be employed in the economy of the body itself, or in changing its place in relation to the external world. In order to illustrate his views of the mode in which the vital force is concerned in animal motion, Liebig has recourse to the various remarkable phenomena of the galvanic battery. By a chemical action going on in one spot between acid and metal, a force is created which circulates through a wire of any extent, and which may be employed in any part of it to produce decomposition of bodies held together by the most energetic affinities, or to overcome the most powerful mechanical resistance. Of the nature of the force which circulates in the wire, of the mode of its propagation, and of the manner in which it operates in producing the separation or union of chemical elements, or the movement of particles or masses of matter, we really know not one iota more, than we know regarding the corresponding manifestations of the vital force.

The nerves are thought by Liebig to resemble the wire of the galvanic circuit in their mode of producing motion:

"By means of the nerves, all the parts of the body, all the limbs, receive the moving force which is indispensable to their functions, to change of place, to the production of mechanical effects. *Where nerves are not found motion does not occur.* The excess of force generated in one place is conducted to other parts by the nerves. The force which one organ cannot produce in itself is conveyed to it from other quarters; and the vital force which is wanting to it, in order to furnish resistance to external causes of disturbance, it receives in the form of excess from another organ, an excess which that organ can not consume in itself." (p. 220.)

The readers of this Journal scarcely require to be reminded, that the doctrine contained in this quotation is one which we have on all occasions felt it our duty to protest against. If it were true, how could the fact be explained, that there is an extensive system of muscles, which can with great difficulty be acted on by any stimulus conveyed through the nerves, whilst they are readily excitable by stimuli applied directly to themselves; or that, in all instances, the nerves lose their power of communicating stimuli after the circulation has ceased, long before the muscles lose their irritability? Equally erroneous is the statement in the former part of the work, that, whilst in the animal organism everything to which the name of *motion* can be applied proceeds from the nervous apparatus, the phenomena of motion in vegetables, the circulation of fluid, for example, in the characeæ, and the closing of the flowers and leaves depend on physical and mechanical causes. (p. 3.) We do not apprehend that any valid distinction can be drawn between the folding of the leaves of the mimosa or the closure of the trap of the dionœa, on the one hand, and the action of the heart and alimentary canal of the highest animal on the other. Both are equally dependent upon stimuli external to them, acting on a contractility inherent in them; and this contractility is, in one case as in the other, a manifestation of vital force. In his next proposition, however, we feel assured that our author is correct; and we have a strong opinion of its importance as a physiological principle:

"We observe further, that the voluntary and involuntary motions, in other words, all mechanical effects in the animal organism, are accompanied by, nay, are dependent on, a peculiar change of form and structure in the substance of certain living parts, the increase or diminution of which change stands in the very closest relation to the measure of motion, or the amount of force consumed in the motions performed. As an immediate effect of the manifestation of mechanical force, we see that a part of the muscular substance loses its vital properties, its character of life; that this portion separates from the living part, and loses its capacity of growth and its power of resistance. We find that this change of properties is accompanied by the entrance of a foreign body (oxygen) into the composition of the muscular fibre (just as the acid lost its chemical character by combining with the zinc;) and all experiment proves that this conversion of living muscular fibre into compounds destitute of vitality, is accelerated or retarded, according to the amount of force employed to produce motion. Nay, it may safely be affirmed that they are mutually proportional; that a rapid transformation of muscular fibre, or, as it may be called, a rapid change of matter, determines a greater amount of mechanical force; and conversely, that a greater amount of mechanical motion (of mechanical force expended in motion) determines a more rapid change of matter. From this decided relation between the change of matter in the animal body and the force consumed in mechanical motion, no other conclusion can be drawn but this, that the active or available vital force in certain living parts is the cause of the mechanical phenomena in the living organism . . . . . . . How, indeed, could we conceive that a living part should lose the condition of life, should become incapable of resisting the action of the oxygen conveyed to it by the arterial blood, and should be deprived of the power to overcome chemical resistance, unless the momentum of the vital force, which had given to it all these properties, had been expended for other purposes?" (p. 221.)

Thus the change of matter, the manifestation of mechanical force, and the absorption of oxygen, are, in the animal body, so closely connected with each other, that we may consider the amount of motion and the quantity of living tissue transformed, as proportional to the quantity of oxygen inspired and consumed in a given time by the individual. The nerves are regarded by Liebig in the light of conductors, which convey the vital force from the muscles, and apply it to the purposes of animal motion; and he thinks it a sufficient explanation of the absence of motor properties in the gelatinous tissues, mucous membranes, &c., to advert to the absence of nerves in these tissues. On this hypothesis, the muscles must be regarded as so many generators of vital force; which, when their product has been removed, die away like an annual that has borne its seed. Fully agreeing with him as to the existence of the connexion which he points out between the action of a muscle and the decomposition of its substance, we yet think that the vital force which is employed to produce the motion, so far from being carried away by the nerve, is expended on the spot; and that the function of the nerve is to convey from its central organ some influence which excites the change, just as the wire proceeding from a galvanic battery excites an ordinary chemical decomposition. We doubt not that this simple and obvious analogy would have been adduced by our author himself, if his mind had not been possessed by those erroneous notions respecting the connexion between nervous agency and muscular movement which still prevail amongst his countrymen.* The gist of his argument, however, is not affected by this error; since his purpose is chiefly to establish that relation between muscular action and the waste of the structure, which we with him regard as indisputable. Of this waste a part is continuous and almost invariable, being dependent upon those regular movements which are necessary to the maintenance of life, such as those of the heart, respiratory muscles, &c.; whilst another portion is altogether regulated in amount by the exercise to which the general muscular apparatus has been subjected. The equilibrium between the supply and the waste of matter can only occur, when the portion separated or expelled in a lifeless form is, at the same instant in which it loses its vital condition, restored in another part. The same arterial blood which brings the oxygen to disintegrate, conveys the fibrin to nourish; and the determination of blood to a particular muscle or set of muscles, which occurs as a consequence of their functional activity, becomes the cause of their increased nutrition. But for this replacement *time* is required; the power of growth is much more limited than the waste; and it may be doubted whether the increase can take place at the same moment at which the disintegration is occurring. In order, therefore, to repair the loss which is occasioned by muscular action, it is necessary that there should be a frequent cessation of that action; and this seems to be the object of that rest which is provided for all the voluntary muscles in *sleep.* In this condition the animal is reduced almost to the level of a plant; for, as in the latter, all the vital force is employed upon the nutritive functions. It is, in the opinion of Liebig, from the absence of nerves, which he regards as conducting away the force generated in the several parts of the system, that the plant derives its capacity of almost unlimited growth, and its power of overcoming the strongest chemical attractions in the substances which serve as its food; for instance, that of the oxygen and carbon in the carbonic acid which it decomposes. In the animal, which is supplied with food already assimilated in chemical nature to its own constitution, the vital force is employed as moving power. "In what form or in what manner the vital force produces mechanical effects in the animal body is altogether unknown, and is as little to be ascertained by experiment as the connexion of chemical action with the phenomena of motion which we can produce with the galvanic battery." But we really know no more than this of the mode in which "a certain something, invisible and imponderable in itself (heat), gives to certain bodies the power of exerting an enormous pressure on surrounding objects; we

* It is not a little remarkable that the Hallerian doctrine should now be most violently opposed in the country of its author; whilst it is most strongly upheld in Britain, especially in the Edinburgh School, which at first most stoutly combated its reception.

know not even how this something itself is produced when we burn wood or coals. So it is with the vital force, and with the phenomena exhibited by living bodies. The cause of these phenomena is not chemical force; it is not electricity nor magnetism—it is a force which has certain properties in common with all causes of motion and of change of form and structure in material substances. It is a peculiar force, because it exhibits manifestations which are found in no other known force." (p. 232.)

In the preceding very beautiful train of speculation, there is one important point left out of view; namely, the *constant* action of the involuntary muscles concerned in the functions of respiration and circulation. However true Liebig's doctrine may be in regard to the necessity of a period of repose for the renewal of the muscular substance which has been disintegrated by action, it obviously applies only to the voluntary muscles; and the nutrition of the heart and of the muscles of respiration must be as continual as their loss. Still his general theory,—*that the source of mechanical power in the organism, is the conversion of living parts into lifeless amorphous compounds*, is not in the least invalidated by such objections; which have reference only to the details of its application.

The doctrine here advanced by Liebig—that the rapidity of transformation of muscular tissue is closely connected with the demand made upon its functional activity—has been previously stated, though in a less precise form, by several physiologists. Thus, in Müller's Prolegomena we meet with the following passages:

"As the excretions are constant, even when the supply of nutriment is stopped, it necessarily follows that a constant decomposition of the substance of the body is essentially connected with life. It cannot, indeed, be otherwise, if it be true, as it has been already proved to be, that the vital force is manifested in an animal only while certain stimuli produce in the living tissues constant material changes, of which the phenomena of life are merely the external signs, just as flame is the appearance resulting from the material changes effected in combustion. The impulse to these material changes is given by respiration." (Baly's Translation, p. 38.)

"Exhaustion ensues when the action of an organ is increased without any external stimulus, if the organic force is not increased at the same time. *It appears, therefore, that the very action of organs produces a change in their composition.* It may be that the constant change which is produced in the organic substance by the action of the arterial blood, and which is as necessary to life as the decomposition of the burning matter is to the phenomenon of combustion, is accelerated or increased by the action of the organ, while the renovation from new nutritive matter does not take place with proportionate rapidity, and can only be effected gradually during rest. Generally, however, the more exertion a man uses, the more active seems to be the decomposition of the matters of his body, and the more need has he for nutriment. Both men and brutes that have died after very violent exertion, as in the instance of a stag hunted to death, undergo putrefaction much sooner than animals bled to death." (Ibid. p. 52.)

The same doctrine, modified by the recent discoveries regarding the independent vitality of the several parts of the organism, has been expressed by Dr. Carpenter in his last work, as shown by the following extract:

"Hence it may be stated as a general law, that the vital activity of the cells (and of the parts produced by their transformation) diminishes in proportion to the prolongation of their life; and this law exactly corresponds with what may be observed in comparing the tissues of different kinds which are present in the same body. For we uniformly find that those in which the most active vital changes are going on (such as the nervous and muscular tissues,) are those in which the duration of the individual component portions is the least, as is shown by the rapidity of the changes of removal and reposition which are continually taking place in them. The converse holds good also. Further, it may be remarked—and this is a matter of much practical importance—that anything which increases the functional activity of any particular tissue, thus causing its cells to live faster, diminishes the duration of their lives, as is shown in the increased demand for nourishment which is set up as a consequence of the continued exercise of the muscular or nervous system, and which, being far greater than can be required for such increase of their amount as results from that exercise, necessarily indicates that a corresponding removal of effete matter resulting from the death of the cells, has taken place." (Principles of Human Physiology, p. 535.)

There is, then, in the living animal body, a constant waste of the muscular substance which constitutes a large proportion of its mass; a part of this waste, occasioned by the action of the heart and respiratory muscles, is independent of the will, and goes on at a nearly equal rate during the period of rest; the other part is entirely dependent for its amount on the degree of exertion which has been required from the voluntary muscles during a given time. The degree of this waste may be measured by the quantity of azotized matter in the urine; since, as will be shown hereafter, this matter is entirely derived from the decomposition of the azotized tissues, of which the muscular must supply by far the largest proportion. Its amount, therefore, is proportional to the mechanical effects produced by an individual in a given time; whether the mechanical force has been employed in voluntary or involuntary motions; whether it has been consumed by the limbs, or by the heart or other viscera. In order to maintain the healthy condition of the body, an equivalent amount of azotized food will be required to restore this waste; and in the adult, a perfect balance will be maintained between the consumption of vital force for supply of matter, and that for mechanical effects. But in the infant, whose nutrition is very active, the mechanical power is very low; the vital force being expended, as in the vegetable, on the increase of the organism. In the old man, on the other hand, there is a larger proportion of mechanical power to the amount of food consumed; but a continual waste is going on. The amount of sleep necessary to restore, in the adult labouring man, the loss which has been sustained by activity, may be stated as seven hours out of the twenty-four. An old man is satisfied with only half this amount of sleep, consequently his expenditure of mechanical power should not be more than half. If an adult man expends more mechanical power than can be restored by seven hours' sleep, or diminishes his quantity of sleep so as not to produce the requisite equilibrium, he grows old prematurely. But an infant at the breast sleeps twenty hours and wakes only four; and all the vital force not expended in mechanical efforts goes to generate new tissue. According to Liebig, if we consider the expenditure of vital force in mechanical effects as counterbalanced, in the adult man, by that which produces the formation of new parts,—the ratio being therefore as 100 : 100,—the ratio of mechanical effects in the infant to the formative effects may be stated as 25 : 250; and in the old man as 125 : 50.

All these effects, however, are greatly modified by temperature. As the vital power of the plant, by which it decomposes the carbonic acid of the air, is dependent upon light, so is the energy of the animal dependent upon heat. For the maintenance of this heat, it is provided with a source within itself; namely, the combination of oxygen with the combustible elements of its structure, or with substances contained in its food. The lower the temperature of the surrounding medium, the more oxygen is introduced into the system for this purpose, the faster does the change of its substance go on, the more force is available for mechanical purposes; consequently, if the supply of food is sufficient, and the system has the power of maintaining its proper standard of heat, a moderate external temperature will be favorable to muscular energy; and this common experience teaches. But if the supply of food be deficient, or the loss of heat by external cooling be greater than that which the system can replace, so that the temperature of the body itself be depressed, then the power to produce mechanical effects is diminished, a condition of sleep ensues, and at last even the vital and involuntary motions cease, so that syncope or death supervenes. The quantity of power available for mechanical purposes is also diminished by causes which prevent a due supply of oxygen to the muscular structure; hence it is that in ascending high mountains, the muscular power is greatly diminished by the rarity of the air. Again, the introduction of easily oxidizable substances into the blood, by the union of which with oxygen the animal temperature is kept up without any disintegration of its tissues, may be imagined to prevent the action of oxygen on the muscular substance, and the consequent liberation of mechanical power. This is considered by Liebig to be the mode in which alcohol increases the animal temperature, whilst it diminishes muscular power. He affirms that it is not passed off by any of the excretions, and that there seems no channel by which it can be got rid of, unless it is thus consumed within the body. We cannot help thinking, however, that his statements on this point are too exclusive; for his assertion that alcohol is not separated in the expired air would seem to be contradicted by ordinary observation, the breath often affording pretty *strong* testimony to a fact which the speech denies; and the experiments of Dr. Percy leave no room for doubt on the matter. Moreover, we feel sure that alcohol must have a specific action on the nervous system; else why the extraordinary modification on the functions of the brain produced by it; and whence the passage of this fluid into the ventricles of the brain, which was proved some years since by the ingenious experiments of Dr. Percy? This is another of the many errors in physiological detail, which will, we fear, prevent the value of the author's labours from being justly appreciated by some of those who possess the capability of discerning them.

In the hybernating animal the temperature of the body falls to a low standard, and the vital energy of the whole system is thereby greatly diminished. Only enough remains to perform the involuntary motions necessary for the support of life; but the waste of the muscular apparatus is checked in the same proportion; and there is little expenditure of material, except of that which goes to keep up the temperature of the body, which is chiefly supported by the combustion of the fatty principles. In other cases the active force of the body may be entirely consumed in producing voluntary mechanical effects, in such wise that no force shall remain available for the involuntary motions. Hence the depressed state of the circulation, which is a familiar consequence of excessive fatigue; and hence the death by syncope of an animal urged to a still longer continuance of muscular exertion. It is remarked by Liebig that, when a stag is hunted to death its flesh becomes uneatable in consequence of the metamorphosis which its muscular tissue has undergone; and the badness of the meat of overdriven cattle is a fact familiar to every one. We have seen, however, that this fact had been previously noticed and correctly applied by Müller.

III. *Food of animals, and its applications in their economy.* Referring to our former report (Br. & For. Med. Rev. No. XXVII, p. 298-308,) for the details contained in the portion of the work which bears on this subject, we shall here give such a brief abstract of our author's views as may enable his general argument to be understood. The food of plants entirely consists of the inorganic binary compounds—carbonic acid, water, and ammonia; and their vital force is employed in the decomposition of these, and in the union of their elements into the peculiar proximate principles which are formed by vegetables. The food of animals consists either of the flesh of animals, resembling their own in composition; or of the proximate principles formed by vegetables. Now it is a recent discovery of the highest importance, which has been entirely substantiated by the analyses of Mulder, that the chief azotized proximate principles of plants, namely, vegetable albumen, fibrin, and casein, not only bear a very strong analogy to the corresponding principles, which enter into the composition of animal bodies, but are absolutely identical with them in the proportion of their elements. Hence the problem of the nutrition of herbivorous animals is much simplified; since it is not requisite to imagine that there is any more elaborate process of conversion exercised upon these articles derived from the vegetable kingdom, than is required for those immediately obtained from the animal body. The digestive process separates the azotized vegetable principles from those which are merely hydrates of carbon, such as starch and sugar; and applies the former to the maintenance of the azotized tissues, whilst the latter are destined for another purpose. All parts of the body which have a decided shape, which form parts of organs, contain nitrogen: water and common fat are the only constituents which are destitute of that element; both these are amorphous and unorganized, and only so far take part in the vital process, as that their presence is required for the due performance of the vital functions. The azotized parts are those most liable to decomposition, and require the most constant regeneration. The materials for this regeneration must, in Liebig's opinion, be entirely and immediately derived from the food; and accordingly animals cannot be supported for any length of time upon matters destitute of nitrogenized constituents; whilst they require a less amount of vegetable substances for their nutrition, in proportion as these abound in such constituents.

The nutritive process in carnivorous animals may be advantageously studied in detail, as affording a simple means of determining the mode in which the elements of the food are disposed of. In the adult animal, the sum of the excretions must equal that of the ingesta, the bulk of the body itself undergoing no change. The fæces of such an animal are dry, and contain only the indigestible parts of its food; and the only proper excrement is that expelled by the urinary passage. This consists, in the carnivorous serpents, of urate of ammonia, which contains only two equivalents of carbon for every equivalent of nitrogen; and in the carnivorous mammalia, of urea, in which there is but one equivalent of carbon to one of nitrogen. But in the flesh which forms the food of these animals, and in that of which their own body is composed, the ratio of carbon to nitrogen is at least as eight to one; consequently the amount of carbon to be excreted by some other channel, is six or seven times that of the nitrogen passed off through the kidney. For this purpose, there is no other channel than the respiratory apparatus,—the skin and lungs; by which the carbon is thrown off as an oxidized product. In this mode the hydrogen of the food is also disposed of. Still, nothing is more certain, than that the carbon, hydrogen, and nitrogen given out, though equal in amount to what is supplied in that form, do not directly proceed from the blood. In the adult animal the food serves to restore the waste of matter; certain parts of its fabric have lost the state of vitality, have been expelled from the substance of the organs, and have been metamorphosed into new compounds which are amorphous and unorganized. Every action in the body involves such a waste. It has been already shown to accompany every exertion of muscular power. Recent discoveries regarding the nature of the secreting process have shown that it takes place in that function to a large amount. And we have no hesitation in extending the doctrine of Liebig from the muscular to the nervous system; and in affirming that every act of sensation, every exertion of voluntary power, every operation of the intellect, every play of the emotions,—in short, every functional exercise of the nervous system, involves a similar passage of nervous tissue from the organized to the inorganic condition. There is, therefore, a constant withdrawal from the blood of the elements which are required to supply this waste and to form the tissues; and the elements thus withdrawn must be supplied by the food, all the nutritious part of which is first converted into blood, and then into organized tissue. After having done its work, the nitrogenized portions, of which no further use can be made in the system, are separated by the kidneys; whilst the remaining carbon and hydrogen are oxidized, so as to liberate the necessary supply of animal heat, and are then carried off through the lungs. We have no reason to believe that, in carnivorous animals, a particle of the flesh or blood once received within the body is thrown off by the excretory process, until it has become the living flesh and blood of the being nourished by it. The secretion of bile and of urine goes on in animals that are in a state of hybernation, or are undergoing starvation; and it has been found that the amount of urea formed by dogs fed upon pure sugar for three weeks was equal to that excreted in the normal condition. The formation of these secretions, therefore, has *no direct* connexion with the ingestion of food, and takes place entirely at the expense of the living tissues which are continually in process of disintegration.

Between these two products, however, there is an important difference. Urine is to be regarded in the light of a simple excretion, having no ulterior purpose in the body, and destined simply to carry off what it would be injurious to retain. On the other hand, bile would seem to have a specific use in the digestive process; and it does not pass off as an excretion, until its state has been completely changed. The latter part of this doctrine is founded on the very startling but positive assertions of Liebig, that the constituents of the bile cannot be recognized in the fæces, at least among carnivorous animals, and that we must suppose the whole of the secreted fluid to be reabsorbed, and its hydro-carbon to pass off by the lungs. We must take the liberty of questioning whether, in the present state of chemical knowledge, such an assertion is justifiable. It is well known that the constituents of the bile are recognized with so much difficulty by chemical tests, and are so readily altered in their character by various reagents, that there has been great difficulty in establishing what its proximate principles really are. We do not, therefore, deem the chemist's assertion as to the absence of biliary matter in the fæces altogether valid; more especially with the obvious fact before us, that their colour, at least, is due to it, as is shown by the effects of obstructions to the flow of bile, occurring in the course of disease, or artifically induced, in decolorizing them. But we can readily imagine that the doctrine of Liebig, as to the reabsorption of the biliary secretion, is more correct when applied to the carnivora, than to omnivorous or herbivorous animals; since in the former the fæces are white and dry; and it is only when they approach the condition of the others (as in the case of the domesticated dog or cat) that the fæces seem tinged with bile. Indeed the supposition appears to be required by the remarkable fact, that, whilst the proportion of elements in the entire mass of blood is equal to that of the average of flesh (the formulæ for both, as Liebig has shown, being the same), the sum of the elements of bile and urine amounts to almost precisely the same; so that there seems to be no superfluous carbon for respiration. All the carbon used for this purpose in the carnivora appears to have passed through their tissues, and to have been separated from the general current of the circulation, by the liver. In the herbivora, however, it is admitted by Liebig that a certain proportion of the elements of bile is discoverable in the fæces; and we shall presently see that in them there is another source for the carbon of respiration. In what manner, or in what state, the elements of the bile are reabsorbed and conveyed to the lungs, we are not informed; they must certainly undergo a change previously to their again entering the current of the circulation; since we know that some of the constituents of the secretion are absolute poisons if retained in the blood, and could not, therefore, be re-introduced into it without injury.

The active movements which are so characteristic of the life of the adult carnivora, and which we see them vainly attempting to perform when in captivity, occasion a waste of matter, which affords an ample supply for the respiratory process. In the young animal, however, the movements are not so energetic; the waste (according to our author) is less; and the maintenance of animal heat could not be provided for, unless more carbon were oxidized than has formed a part of the tissues of the body. It is for this purpose that, in the milk which serves for its nutrition, there is not only a supply of azotized matter, the caseine,— but also a store of matter rich in hydrogen and carbon, the buttery and saccharine portions of it, for the purpose of being oxidized in such a manner as to generate the requisite amount of caloric. We cannot but think, however, that there is here an imperfection in the mode of our author's reasoning, although we agree in the general correctness of its result. Upon his own frequently-repeated statement, the proportion of urea excreted is an accurate measure of the waste of the tissues; and this proportion is, with reference to the bulk of the body, much greater in the child than in the adult, as Lecanu's observations have proved. The facility with which the effects of injuries are repaired is another indication that the processes of disintegration, as well as those of renewal, are active in children; since the reparative process often requires not merely the formation of new parts, but the removal of those that have become effete. We are inclined to believe, therefore, that the change of matter goes on *more,* instead of *less,* rapidly in the young animal than in the adult. But its loss of heat, in consequence of the much larger surface it exposes in proportion to its bulk, is *much* greater than that of the adult; and it is to provide against this, that Nature has superadded to the calorific means possessed by the adult a store of combustible matter for the use of the tender nursling. The question may not seem to all our readers to be important enough to sanction this digression; but we hope we shall not be suspected of a captious spirit in stopping to correct physiological errors, even of a trifling kind, whereever they are likely to be preferred to truth, through the influence of so high an authority.

In the herbivorous animal, the process of nutrition is represented by Liebig as very different from that which has been described in the adult carnivora, and as more nearly resembling that which takes place in the young of the latter group. From the comparative indolence of their habits, the waste of their tissues is far less, and the amount of azotized nutriment which they require to support it diminishes in the same proportion. Hence the quantity of carbon thus set free for the maintenance of the heat of the system, is not nearly sufficient for the purpose. But Nature has provided the requisite supply in their food. Whilst the nitrogenized constituents of the food of the carnivorous animal afford, when they return to the state of dead matter after having passed through the living tissues, all or nearly all the carbon required for respiration, those which are sufficient to maintain the normal structure of the herbivora do not yield above one fifth of the carbon which is consumed for the purpose of generating caloric; but the sugar, starch, and other non-azotized principles taken in as food appear to be made *directly* subservient to this function, without ever becoming part of the living fabric. Hence an animal thus constituted, if restricted to a diet consisting entirely of azotized substances, must be supplied with five times the amount of these that would otherwise be necessary; and the very instructive fact is pointed out by Liebig, that since 15 lbs. of flesh contain no more carbon than 4 lbs. of starch, a savage, with one animal, and an equal weight of starch, could support life for the same length of time, in which another restricted to animal food would require five such animals, in order to procure the carbon necessary for respiration. When confined to animal food, man is made to resemble the carnivora in his nutrition; and as, like them, he derives the carbon for his respiration from the waste of his tissues, like them he is obliged to expedite that waste by continual activity. Cultivation, therefore, by which a large amount of non-azotized matter is supplied for respiration, is not merely the economy of nutrition, but the economy of force. This statement requires some modification, however, when it is applied to races of men like the Esquimaux, who, though restricted to animal food, are chiefly supported upon that which resembles the non-azotized elements of plants, in its capacity for being at once oxidized, and for thereby sustaining the temperature of the system; for all the fatty constituents of the fabric must be regarded as serving this purpose; and these abound in the animals upon which such races feed. Hence the restless activity which is observable among the hunters of the agile deer or the swift-footed antelope of southern climes, gives place among the inhabitants of the frigid zone to a stupid inertness, which is only occasionally interrupted by the necessity of securing the supply of food afforded by the massive tenants of their seas. Here, as in other instances, the exception adds probability to the rule; and we see, in the kind of food most readily obtainable by the inhabitants of different regions, a beneficient provision of the Creator for their respective wants.

Since the preceding paragraph was written, our attention has been directed to the fact that the portion of Liebig's doctrines, which concerns the direct application of the non-azotized elements of the food to the maintenance of animal heat by a species of combustion, has been long taught in this country, by Dr. Collier. In the surgeon's Report, appended to Sir John Ross's Narrative of his Arctic Voyage, (published in 1835), which Report was written by Dr. Collier from data verbally supplied by the surgeon, we find the following passages.—" If the preservation of a uniform temperature by external means be of the highest importance, it must be admitted that the due and vigorous generation of caloric by a proper selection of food is not less so. The natural food of this climate seems well adapted to the purpose. Every one knows that solar caloric, caloric by combustion, and that generated by animal life, are the three chief sources by which our temperature is sustained. Now, it seems but reasonable that in a region where our supply from the first two is so exceedingly limited, the more active evolution from the last source should compensate for the deficiency..... There are three modes by which heat is probably generated within the body, by the chemical decomposition which takes place in respiration, by the influence of the brain and nervous system, in some degree analogous, perhaps, to its development by galvanic influence, and by the process of digestion and nutrition. If it be acknowledged that combustion goes on more rapidly in cold weather, and that this is wisely pre-ordained, the same remark applies to respiration, in which the imaginative poet and the philosopher alike recognize the resemblance. The heat generated will partly depend on the rapidity of union of the impurities of the blood and the consequent liberation of caloric. *But it will partly depend on the quantity of carbon and hydrogen contained and taken in with the food.* On this ground alone, I expect the patience of my readers; for it will follow, if this be admitted, that such provisions should be selected for these expeditions as may have been found to contain these elements in the largest possible excess, loosely combined, and in the most favorable state for elimination. On reference to the food destined by nature for the support of the Esquimaux, we find it almost exclusively hydro-carbonaceous oil, blubber, fish, and flesh, the latter two of which cannot be too fat for them. Here we see a strong analogy between their process of nutrition and that of combustion; nearly the same materials, the same play of affinities, the same results, the same change of latent into sensible caloric. If I am rightly understood, my readers must see that I contend that the gross diet of northern tribes is not a matter of chance, but in harmony with the slow but constant changes which are continually going on around them; and intended to enable them to resist cold, and to vigorously generate heat." (Appendix to Sir J. Ross's Voyage, pp. cxxv-cxxvii.)

We do not think that Liebig would lay any great stress upon this doctrine as peculiar to himself. The point on which he evidently most insists, is the subserviency of the carbon of the bile to the maintenance of animal heat by the respiratory process; as evinced by its re-absorption, and disappearance through that channel. We doubt not that scattered hints could be found, in the writings of chemists and physiologists long anterior to Dr. Collier, respecting the use of the hydro-carbon of the food in maintaining animal heat through respiration; since the analogy of this process to that of combustion has been, as Dr. C. himself remarks, long generally recognized.

The slowness of the metamorphosis of the tissues of the herbivora is considered by Liebig as sufficiently indicated by the very small amount of the alkaline phosphates contained in their urine; since the presence of phosphorus appears essential to the existence of fibrin and albumen as such, and it must therefore be set free when they are decomposed. But they lose much more heat than the carnivora, in consequence of the large amount of perspiratory glands in their skin; and therefore, whilst they require far less azotized matter for their food, they have need of much more carbon and hydrogen. But if the process of cooling and exhalation be checked, as it is in stall-fed animals, the non-azotized constituents, instead of being thrown off by respiration, in the form of carbonic acid and water, are retained in the system, and metamorphosed into *fat*. This transformation is effected simply by the withdrawal of a part of the oxygen; and as the change of the azotized principles into fat would be a much more complex process, it may be safely assumed that this product is formed from sugar, starch, and other substances of the same kind. We have in the bee, as correctly stated by Liebig, a very satisfactory example of the transformation of a saccharine into a fatty body; since it has been demonstrated that wax may be formed, to any amount, when bees are fed upon pure sugar. A deposit of fat will take place, therefore, whenever the amount of carbon in the food is greater than can be carried off by the oxygen introduced by respiration; and this may be due to a superabundance of carbon, or to a deficiency of oxygen. Anything which increases the supply of oxygen, such as bodily exercise, will tend to keep down the accumulation. But in the very production of fat, oxygen is set free in the form of carbonic acid; and this process itself becomes a source of the disengagement of heat.

The doctrine thus expounded enables us to give a much more precise account of many pathological changes which could only be previously explained in a vague and general manner. Thus, when the respiratory process is permanently interrupted by chronic disease of the lungs, the condition is produced which is favorable to the generation of fatty matter; there seems, however, to be a general condition of the system which is unfavorable to its deposit in the tissues; and the office of separating it is then thrown on the liver, which, as Mr. Bowman has recently shown, has a tendency to present the fatty condition, simply from being called upon to separate an unusual quantity of fatty matter from the blood. Again, in some other diseases, according to Liebig, the starch, sugar, &c., of the food obviously do not undergo the changes which enable them to assist in respiration, and consequently to be converted into fat; thus in diabetes mellitus, the starch is only converted into grape sugar, which is expelled from the body without further change. We are not sure, however, that this explanation of the disease is not too directly chemical; and we are more inclined to take Dr. Prout's view of its nature, in referring it to a disordered condition of the process of decomposition of the tissues at large. The craving appetite, which is so remarkable a symptom in an early stage of it, and which generally continues through its whole course, is a sufficient indication to our minds that the *waste* (as it is termed by Liebig) is going on with unusual rapidity; and the enormous amount of the azotized constituents of food which can be appropriated without bodily exercise, seems to show that there is something wrong in the process of their assimilation, as well as in the treatment of the non-azotized compounds. The influence which the total withdrawal of sugar, starch, &c. from the food has in controlling the disease, is certainly in favour of Liebig's view; but, on the other hand, if it were altogether correct, the formation of sugar ought to cease entirely as soon as the supply of non-azotized matter is stopped, which is certainly not the case; and it ought to bear a constant proportion to the supply of non-azotized matter, which is also far from being true, as the practical physician well knows. We do not believe that we are yet in a condition to understand the real nature of this interesting disease; and we would put our readers on their guard against accepting the dogmas even of a Liebig with respect to it, especially since (as we shall presently find) he entertains a conception which has been *proved* to be erroneous. The practical physician well knows, that the excretion of sugar by the urine,—the point which is naturally most striking to the chemist,—is really but a small part of the whole matter.

This division ends with a few observations on the part performed by gelatine in nutrition. That principle is regarded by Liebig as entirely incapable of affording the means of reparation to the fibrous and albuminous tissues; but he considers that, when taken into the system, it may serve to replace the waste of the gelatinous tissues themselves,—cartilage, bone, cellular membrane, &c. A certain power must be requisite, in the state of health, to form such from the fibrin or albumen of the blood; hence it may be considered as an economy of this power to employ a certain amount of ready-prepared gelatine as food; and this economy will be the more decided, when the body has been enfeebled by sickness. But all experience shows that on gelatine alone the system cannot be supported.

IV. *Metamorphosis of tissues.* Under this head our author combines a large number of highly interesting facts relating to the changes to which the alimentary principles are subservient within the body. He by no means intends to offer a complete history of these changes; but simply to indicate the path of inquiry by which their nature may be elucidated. He commences by pointing out the important fact, that experiment has now demonstrated the existence of numerous compounds of a simply chemical nature, which, with the greatest diversity in external characters, possess the same composition in 100 parts, or even the same absolute amount of equivalents in each element.

"Cyanuric acid, for example, is a nitrogenized compound which crystallizes in beautiful transparent octahedrons, easily soluble in water and in acids, and very permanent. Cyamelide is a second body, absolutely insoluble in water and acids, white and opaque, like porcelain or magnesia. Hydrated cyanic acid is a third compound, which is a liquid, more volatile than pure acetic acid, blisters the skin, and cannot be brought in contact with water without being instantaneously resolved into new products. These three substances not only yield, on analysis, absolutely the same relative weight of the same elements, but they may be converted and reconverted into one another, even in hermetically closed vessels; that is, without the aid of any foreign matter." (p. 104.)

A similar group of three occurs in the case of albumen, fibrin, and casein. When these substances are dissolved in a moderately-strong solution of caustic potash, and the solution is exposed for some time to a high temperature, they are decomposed. The addition of acetic acid to the solution causes in all three the separation of a gelatinous translucent precipitate, which has exactly the same characters and composition, from whichever of the three substances above mentioned it has been obtained. This compound has been termed *protein* by Mulder, its discoverer; and he has found by precise and careful analysis, that it contains exactly the same elements and exactly in the same proportion as the animal matters from which it is prepared; whilst these last may be regarded as compounds of proteine with definite proportions of sulphur and phosphorus. The same product may be obtained from the azotized principles of plants. Hence we may take this as a common foundation or point of departure in investigating the metamorphoses which occur in the animal body; since all its organic nitrogenized constituents, how different soever in composition, may be regarded as formed from proteine by the addition or subtraction of the elements of water or of oxygen, and by resolution into two or more compounds. Of this fact the development of the chick *in ovo* may be considered a sufficient demonstration; since the egg contains nothing but albumen, with a small proportion of fatty matter. The carbon of the latter may partly contribute to form the nerves and brain, of which this element constitutes a large proportion, and is partly thrown off by respiration; whilst the fibrin, blood-globules, muscles, vessels, membranes, feathers, claws, &c. cannot be formed in any other way than from the albumen.

We think it necessary to point out, however, that in various passages of his work, a want of acquaintance is shown by Liebig as to what we think may be considered to be the true relations of albumen and fibrin. The former we regard as a mere chemical compound, incapable of undergoing organization, until its condition has been entirely changed, and possessing only a granular structure, when it is made to coagulate. The latter, on the other hand, we believe to be the *pabulum*, at the expense of which, not only muscular fibre, but all the tissues of the protein series are immediately formed; it possesses truly vital properties, as evinced by its tendency to assume a regularly-organized form, when it coagulates in a thin layer on a living surface (as in organizable lymph); and it may altogether be considered as the intermediate form between albumen and the living tissues.

The process of chymification is viewed by Liebig as a purely chemical action, analagous to those processes of transformation which were described in his former work as putrefaction, fermentation, and eremacausis. These changes depend upon the influence which an azotized substance, itself in a state of transformation, is capable of exerting upon other matters whose constituents are held together somewhat loosely.

"The clear gastric juice contains a substance in a state of transformation, by the contact of which with those constituents of the food which, by themselves, are insoluble in water, the latter acquire, in virtue of a new grouping of their atoms, the property of dissolving in that fluid................ It can hardly be doubted that this substance is a product of the transformation of the stomach itself. No substances possess in so high a degree as those arising from the progressive decomposition of the tissues containing gelatine or chondrine, the property of exciting a change in the arrangement of the elements of other compounds. When the lining membrane of the stomach of any animal—as, for example, that of the calf—is cleaned by continual washing in water, it produces no effect whatever if brought into contact with a solution of sugar with milk, or other substances. But if the same membrane be exposed for some time to the air, or dried and then placed in contact with the same substances, the sugar is changed according to the state of decomposition of the animal matter, either into lactic acid, into mannite and mucilage, or into alcohol and carbonic acid; while milk is instantly coagulated. An ordinary animal bladder retains, when dry, all its properties unchanged; but when exposed to air and moisture it undergoes a change not indicated by any obvious external signs. If, in this state, it be placed in a solution of sugar of milk, that substance is quickly changed into lactic acid." (p. 110.)

"The fresh lining membrane of the stomach of a calf, digested with weak muriatic acid, gives to this fluid no power of dissolving boiled flesh or coagulated white of egg. But if previously allowed to dry, or if left for a time in water, it then yields, to water acidulated with muriatic acid, a substance in minute quantity, the decomposition of which has already commenced and is completed in the solution. If coagulated albumen be placed in this solution, the state of decomposition is communicated to it, first at the edges, which become translucent, pass into a mucilage, and finally dissolve. The same change gradually affects the whole mass, and at last it is entirely dissolved, with the exception of fatty particles, which render the solution turbid." (p. 111.)

It is scarcely possible, in our opinion, for any demonstration to be clearer; and if anything were wanting to confirm it, it is the fact recently established by the labours of German physiologists, that the whole epithelium of the stomach is thrown off at every meal, thus furnishing the very supply of animal matter in a state of decomposition which is theoretically required. In the action of the gastric juice on the food, the oxygen of the atmosphere and the elements of water take an important share; and it is considered by Liebig that the chief use of the saliva is to entangle and carry down, by means of its peculiar viscidity, a large quantity of air-bubbles to aid in digestion. The nitrogen of the air thus introduced is believed by him to be taken into the blood, and to be the source of that which is exhaled from the skin and lungs. We cannot but regard this supposition as rather far-fetched, and as founded on somewhat insufficient evidence; still we have no ground for denying it; and any account of the peculiar objects of the salivary secretion has the merit of assigning a purpose for that which is evidently an important function, but which has never yet been sufficiently elucidated.

The exact state and proportion in which sulphur and phosphorus exist in fibrin and albumen has not been exactly determined; and Liebig is not sanguine in his expectations as to the possibility of any such determination; since a variation in the sulphur or phosphorus, smaller in extent than the usual limit of errors of observation, will affect the number of atoms of carbon, hydrogen, or oxygen, to the extent of ten atoms or more. "A formula for protein," he further remarks, "is nothing more than the nearest and most exact expression in equivalents of the result of the best analyses; it is a fact established so far, free from doubt, and this alone is for the present valuable to us." This formula is 48 C, 6 N, 36 H, and 14 O. In albumen the proportionals of sulphur and phosphorus are equal; in fibrin the amount of sulphur is double that of the phosphorus; whilst in casein there is no phosphorus, but only sulphur. The following table represents the chemical alterations to which the protein is subjected in its transformation into various organized tissues:

| | *Protein.* | *Ammonia.* | *Water.* | *Oxygen.* |
|---|---|---|---|---|
| Fibrin, Albumen | Pr. | . . . | . . | . . |
| Arterial membrane | Pr. | . . . | + 2 H.O. | . . |
| Chondrine . | Pr. | . . . | + 4 H.O. | + 2 O. |
| Hair, Horn . | Pr. | + $N.H_3$ | . . | + 3 O. |
| Gelatinous Tissues | 2 Pr. | + 3 $N.H_3$ | + H.O. | + 7 O. |

"From this general statement it appears, that all the tissues of the body contain, for the same amount of carbon, more oxygen than the constituents of the blood. During their formation oxygen also, either from the air or from the elements of water, has been added to the elements of protein. In hair and gelatinous membrane we observe, further, an excess of nitrogen and hydrogen, and that in the proportions to form ammonia.

"Chemists are not yet agreed on the question, in what manner the elements of the sulphate of potash are arranged: it would therefore be going too far, were they to pronounce arterial membrane to be a hydrate of protein; chondrine, a hydrated oxide of protein; and hair and membranes, compounds of ammonia with oxides of protein." (p. 127.)

The greatest difficulty in the comprehension of these transformations is connected with the production of gelatinous and horny substances. To imagine that, in their formation, ammonia as well as oxygen and water are added to the protein, appears a very improbable supposition; and the idea that they are elaborated from protein chiefly by a disengagement of certain equivalents of carbon and hydrogen, appears much more consistent with the established fact, that gelatine and its allied principles are entirely removed from the compounds of the protein series, and never can be reconverted into them. That a separation of carbon takes place in the conversion of fibrin or albumen into gelatine, was long ago suggested by Dr. Prout; and he indicated this process, which must be continually going on, as one source of the carbon which is set free in the lungs by the function of respiration—an idea which would now seem to require modification.

If the view propounded by Liebig with regard to the respiratory process—that the carbon and hydrogen set free by it in an oxidized form are rather derived from the reabsorption of bile, or from the non-azotized portion of the food, than immediately from the decomposition of the tissues—should prove correct, we should expect that the two principal excretions, the bile and urine, would contain nearly all the products of the waste of the system ; and that the sum of their constituents should be equivalent to the sum of the elements of the living flesh, or of the circulating blood, which has been well designated as liquid flesh (*chair coulante*). However ill founded such an expectation might seem, the analyses of Liebig and his associates leave us no room for doubt on the subject; and to his general theory we can scarcely refuse our assent, although when it comes to be applied to other classes of animals, we apprehend that it will need considerable modification. Thus in insects, whose respiration is so peculiarly energetic when they are in a state of activity, we find but the merest traces of a biliary apparatus; and the mode in which atmospheric air is introduced into the minutest portions of the body, together with the extraordinary increase in the quantity of carbon thrown off during violent muscular movement, leads us to think that in this class very nearly the whole carbon thrown off by respiration is at once derived from the decomposition of the tissues, and does not pass through the biliary apparatus at all. On the other hand, the mollusca and crustacea, which agree in aquatic residence, and in inertness of habits, agree also in possessing an immense liver, combined with a very feeble respiratory apparatus; and we can scarcely avoid the belief that in them more of the carbonaceous matter of the bile passes off with the excrements, than in warm-blooded animals, where it is needed for the support of the temperature.

The analyses of Playfair and Boeckmann give for flesh (fibrin, albumen, cellular tissue, and nerves,) and for blood, as the most exact expression of their numerical results, one and the same formula, namely, 48 C, 6 N, 39 H, 15 O. This may be called the empirical formula of blood. Now the most characteristic ingredient of bile appears to be a substance of an acid nature, termed *choleic* acid, which is capable of being readily converted into other forms (such as *choloidic* acid) by the operation of various agents. The results of its analysis are best expressed by the following empirical formula; 76 C, 2 N, 66 H, 22 O. Thus we see that a predominance of carbon is the peculiar character of the biliary secretion. Now if we add to half this formula the elements of the urate of ammonia, which is the characteristic ingredient of the urine of serpents, we get as the sum the following formula: 48 C, 6 N, 40 H, 17 O, which is exactly that of blood, with the addition of one equivalent of water and one of oxygen—the very elements which we have independent reason to know are added in the course of the transformations in question. In the higher classes of animals uric acid is no longer found in the urine, but is replaced by urea. This disappearance of uric acid and production of urea plainly stand in very close relation to the quantity of oxygen absorbed in respiration and to the quantity of water consumed in a given time; for when uric acid is subjected to the action of oxygen it is at first resolved, as is well known, into urea and alloxan; whilst a further supply of oxygen acting on the alloxan causes it to resolve itself into urea and either oxalic or carbonic acid, the latter being formed when there is most oxygen. Hence it is remarked by Liebig, that the tendency to the formation of calculi consisting of uric acid will be greatest in those in whom, from want of exercise, the supply of oxygen is deficient; if in such cases the amount of exercise be increased, oxalates will be formed instead of urates—a result which he affirms to be conformable to experience. With a still greater supply of oxygen the same elements would have yielded in healthy subjects only the last product of the oxidation of uric acid, namely, carbonic acid and urea. The action of oxygen upon uric acid is favoured by the ingestion of a large quantity of water, by means of which the sparingly soluble uric acid is kept dissolved, so that the inspired oxygen can act upon it: in birds, which drink but little, uric acid predominates in the urine, in spite of their active respiration. In the urine of the horse when at rest, hippuric acid is found; and this is converted, by increased oxygenation, into benzoate of ammonia and carbonic acid as soon as the animal is compelled to labour. In the fœtal calf the urine contains a peculiar product—allantoin; and Liebig points out that two proportionals of protein, with the addition only of two atoms of water, contain the elements of six proportionals of allantoin and one of choloidic acid, which seems to be the chief constituent of the meconium. These six atoms of allantoin contain the elements of two atoms of uric acid, two atoms of urea, and two atoms of water. So that in the fœtal calf, as in the former instances, the urinary and biliary secretions together account for those elements which were once united in the protein of the tissues.

Such comparisons further lead to a curious view of the connexion between the formation of these secretions, and the metamorphoses of protein into gelatine. For if we suppose one of three atoms of protein to be decomposed into its nitrogenized products urea and uric acid, and the non-azotized choloidic acid, and consider the latter to be withdrawn, whilst the former remains united with the other two atoms of protein, we obtain a formula closely approaching to the composition of gelatinous tissues. In regard to the speculations as to the connexion between the secretion of bile and the formation of gelatine, to which we might be thus conducted, our author expresses himself with philosophic caution:

"We must, however, attach to such formula, and to the considerations arising from them, no more importance than justly belongs to them. I would constantly remind the reader that their use is to serve as points of connexion, which may enable us to acquire more accurate views as to the production and decomposition of those compounds which form the animal tissues. They are the first attempts to discover the path which we must follow in order to attain the object of our researches; and this object, the goal we strive to reach is, and must be, attainable. The experience of all those who have occupied themselves with researches into natural phenomena leads to this general result—that these phenomena are caused, or produced, by means far more simple than was previously supposed, or than we even now imagine; and it is precisely their simplicity which should most powerfully excite our wonder and admiration." (p. 143.)

The preceding remarks on the bile have reference only to the Carnivora, in which alone its whole solid matter can be traced to the metamorphoses of tissues; in the Herbivora there seems good reason to believe, that a large part of the non-azotized compounds which abound in it are derived immediately from the corresponding principles of their food. Thus it has recently been pointed out by Berzelius (though we do not find the fact adverted to by Liebig) that the green colouring matter of the bile of ruminants seems identical with the chlorophyll of the herbage on which they feed. The transformation of the animal tissues will be probably influenced by the presence of such other substances; just as, according to the interesting experiments of Dr. A. Ure, the administration of benzoic acid causes the usual constituents of the urine to be replaced by hippuric acid. Thus Liebig shows that if the elements of starch and protein, oxygen and water being also present, undergo transformation together and mutually affect each other, we obtain, as the products of this metamorphosis, urea, choleic acid, ammonia, and carbonic acid, and besides these no other product whatever. He then goes on to point out some very curious relations between the azotized constituents of the bile and those of the urine; which in his opinion show that the azotized products of the transformation of tissues in the herbivora do not, as in carnivora, reach the kidneys immediately or directly; but that, before their expulsion from the body in the form of urine, they take a share in certain other processes, especially in the formation of the bile; after which they are reintroduced into the circulation, and are not finally carried out of it by the urine until they have undergone their final metamorphosis. There would seem to be in the herbivora a sort of economy of azotized matter, arising out of the small degree of waste of their tissues, and the small amount of azotized principles which are taken into the system to replace this. The opinion that the non-azotized compounds of the food at once pass off by the various channels of excretion, especially the liver, is regarded by Liebig as confirmed by the fact that not a trace of starch or sugar has been detected in arterial blood, not even in animals which had been fed exclusively with these substances; but we are inclined to question the truth of this assertion, since it is succeeded by one that has been proved to be erroneous,—the total absence of sugar in the blood of diabetic patients. It is well known that the presence of sugar in diabetic blood has been of late established, not only by chemical tests, but by the perhaps still surer agency of polarized light.

v. *Influence of medicinal agents on vital transformations.* The statement of Dr. A. Ure, already referred to, respecting the influence of benzoic acid taken into the system in producing hippuric acid, has received satisfactory confirmation from the experiments of M. Keller, in the laboratory of Professor Wohler at Göttingen, as well as in this country by Mr. Garrod. These experiments are considered by Liebig as placing beyond all doubt the fact "that a non-azotized substance taken in the food can take a share, by means of its elements, in the act of transformation of the animal tissues, and in the formation of a secretion." If this principle be admitted, it at once affords us a key to the *modus operandi* of a great number of remedies; and, although the details of their action have not yet been investigated, it is a great thing to know in what direction to look for them. We consider this portion of Liebig's work as the one which is of the highest value to the scientific pathologist, although extremely scanty in its details. In fact, the purpose of the author is evidently to give but examples of the kind of investigation which he proposes; examples, however, which are in themselves of the greatest interest, as well as of the highest promise. Reverting to the speculations which he has previously offered as to the sources of the biliary secretion in the herbivorous animals, he shows that the presence of a nitrogenized compound is essential to the formation of that fluid; and that this compound, like the other constituents, may be obtained directly from the food, instead of being produced by the metamorphosis of tissues. Now, it is among the most curious results of late analyses that *theine*, the peculiar principle of tea, should prove to be identical in composition with *caffeine*, the active principle of coffee; and that both these should contain the same proportions of carbon and nitrogen as exist in *taurine*, the azotized principle of bile, so as to be convertible into it by the addition of water and oxygen. The same may be said of asparagine, the characteristic principle of asparagus and althæa; and by a similar addition of water and oxygen to theobromine, the characteristic principle of the cocoa bean, it will yield the elements of taurine and urea, of taurine, carbonic acid, and ammonia,—or of taurine and uric acid. However small the quantity of solid matter contained in an infusion of tea or coffee, still it will be sufficient to form a corresponding amount of the azotized ingredient of bile, without which those ingredients that constitute by far the largest portion of the secretion will not be separated. In the natural condition of man such artificial aids are not required. But it cannot be denied that,—

"In the case of an excess of non-azotized food, and a deficiency of motion (which is required to cause the change of matter in the tissues, and thus to yield the nitrogenized product which enters into the composition of bile), the health may be benefited by the use of compounds which are capable of supplying the place of the nitrogenized substance produced in the healthy state of the body, and essential to the production of an important element of respiration. In a chemical sense,—and it is this alone which the preceding remarks are intended to show,—caffeine or theine, asparagine, and theobromine are, in virtue of their composition, better adapted to this purpose than all other nitrogenized vegetable principles. The action of these substances, in ordinary circumstances, is not obvious, but it unquestionably exists." (p. 182.)

Passing from these to the truly medicinal agents, it is remarked that these may be subdivided into three groups: the first (including the metallic poisons) consists of substances which enter into direct chemical combination with certain parts or constituents of the body, while the vital force is insufficient to destroy the compounds thus formed; the second, consisting of the essential oils, camphor, empyreumatic substances, antiseptics, &c., possesses the property of impeding or retarding those kinds of transformation to which certain very complex organic molecules are liable, transformations which, when they take place out of the body, are usually designated by the terms fermentation and putrefaction; the third division is composed of bodies the elements of which take a direct share in the changes going on in the animal body, although not furnishing materials for the formation of blood, and therefore not, strictly speaking, nutritive in their character. It is with this last group alone that we are at present concerned; and the example just given affords a beautiful illustration of the mode in which an important process of *secretion* may be influenced by the ingestion of substances which cannot be converted into blood. There is no reason why the *nutrition* of certain parts which differ widely from the protein compounds in the proportion of their elements, should not be influenced in like manner; and this would seem by far the most probable view of the *modus operandi* of certain medicines, which have a specific and direct effect upon the nervous system. The nitrogenized vegetable principles, whose composition differs from that of the proper nitrogenized elements of nutrition also produced by the vegetable organism, are distinguished, beyond all others, for their powerful action on the animal economy. The effects of these substances are singularly varied; from the mildest form of the action of aloes, to that most terrible poison, strychnia, we observe an endless variety of different actions. It is asserted by Liebig that no substance devoid of nitrogen possesses a poisonous action in a similar dose; and he states that from this suggestion M. Francis was led to examine more accurately the composition of picrotoxine, the active principle of cocculus indicus, and to discover in it the presence of nitrogen which had been previously overlooked. It is certainly a most valuable test of the validity of a law, that it is able to correct observations which appeared to contradict it. Now the primary operation of these powerful agents is universally referred to the nervous system.

"This action is commonly said to be dynamic, that is, it accelerates, or retards, or alters in some way the phenomena of motion in animal life. If we reflect that this action is exerted by substances that are material, tangible, and ponderable—that they disappear in the organism—that a double dose acts more powerfully than a single one—that after a time a fresh dose must be given if we wish to produce the action a second time; all these considerations, viewed chemically, permit only one form of explanation; the supposition, namely, that these compounds, by means of their elements, take a share in the formation of new, or the transformation of existing brain and nervous matter.

"However strange the idea may at first sight appear, that the alkaloids of opium or of cinchona bark, the elements of codeine, morphia, quinine, &c., may be converted into constituents of brain and nervous matter, into organs of vital energy, from which the organic motions of the body derive their origin; that these substances form a constituent of that matter, by the removal of which the seat of intellectual life, of sensation, and of consciousness is annihilated; it is, nevertheless, certain that all these forms of power and activity are most closely dependent, not only on the existence, but also on a certain quality of the substance of the brain, spinal marrow, and nerves; insomuch that all the manifestations of the life or vital energy of these modifications of nervous matter, which are recognised as the phenomena of motion, sensation, or feeling, assume another form, as soon as their composition is altered. The animal organism has produced the brain and nerves out of compounds furnished to it by vegetables; it is the constituents of the food of the animal which, in consequence of a series of changes, have assumed the properties and the structure which we find in the brain and nerves." (p. 183.)

It is not, then, more improbable that the formation of the nervous matter should be influenced by the ingestion of azotized vegetable principles of one kind, than that it should be entirely dependent, as we know that it normally is, on the ingestion of other principles furnished by the same kingdom. But why, it may be asked, should we seek for such an influence rather in the nervous tissue than in any other? The reply furnished by Liebig is pregnant with meaning. Of all the tissues in the body, the matter of the nerves is that to which these powerful principles bear the nearest resemblance in chemical composition. We have seen that vegetable fibrin, albumen, and casein are transformed into muscle and other fibrous tissues, because there is no essential difference in their chemical composition. We have seen that the starch and sugar of plants are transformed, under certain conditions, into the fat of animals ; the required chemical change being one easily comprehended. Now, the peculiar matter of the nervous tissue is of a nature chemically intermediate between protein and fat; for it is a fatty acid, containing a small proportion of nitrogen, and must be formed either by the separation of a highly-azotized compound from the elements of the blood, or by the combination of a nitrogenized product of the vital process with a non-azotized compound, probably a fatty body. This process of conversion is probably not a simple one, but consists of several stages, in each of which a protein-compound undergoes a certain alteration. It is easy to conceive, then, that an azotized principle introduced from without may take the place of these altered compounds; just as we have reason to believe that the ingestion of ready-prepared gelatine may supersede the necessity of its formation in the system, or that caffein may supply the nitrogenized constituent required for the bile. And thus we have what is, to say the least, a feasible explanation of the powerful action of minute quantities of these vegetable principles upon the nervous system.

"It would serve no purpose to give these considerations a greater extension at present. However hypothetical they may appear, they only deserve attention in so far as they point out the way which chemistry pursues, and which she ought not to quit, if she would really be of service to physiology and pathology. The combinations of the chemist relate to the change of matter, forwards and backwards, to the conversion of food into the various tissues and secretions, and to their metamorphosis into lifeless compounds; his investigations ought to tell us what has taken place and what can take place in the body. It is singular that we find medicinal agencies all dependent on certain matters, which differ in composition; and if, by the introduction of a substance, certain abnormal conditions are rendered normal, it will be impossible to reject the opinion that this phenomenon depends on a change in the composition of the constituents of the diseased organism, a change in which the elements of the remedy take a share; a share similar to that which the vegetable elements of food have taken in the formation of fat, of membranes, of the saliva, of the seminal fluid, &c. Their carbon, hydrogen, or nitrogen, or whatever else belongs to their composition, are derived from the vegetable organism ; and after all, the action and effects of quinine, morphia, and the vegetable poisons in general, are no hypotheses.

"Thus, as we may say in a certain sense, of caffeine, or theine and asparagine, &c., as well as of the non-azotized elements of the food, that they are food for the liver, since they contain the elements by the presence of which that organ is enabled to perform its functions, so we may consider these nitrogenized compounds, so remarkable for their action on the brain, and on the substance of the organs of motion, as elements of food for the organs as yet unknown, which are destined for the metamorphosis of the constituents of the blood into nervous substance and brain. Such organs there must be in the animal body; and if, in the diseased state, an abnormal process of production or transformation of the constituents of cerebral and nervous matter has been established ; if, in the organs intended for this purpose, the power of forming that matter out of the constituents of the blood, or the power of resisting an abnormal degree of activity in its decomposition or transformation, has been diminished; then, in a chemical sense, there is no objection to the opinion, that substances of a composition analogous to that of nervous and cerebral matter, and consequently adapted to form that matter, may be employed, instead of the substances produced from the blood, either to furnish the necessary resistance, or to restore the normal condition." (pp. 188-9.)

It is a strong confirmation of any doctrine, to find several inquirers arriving at the same result by entirely different paths of investigation. Thus we have seen that Liebig has been led, by inferential evidence of an entirely chemical nature, to the conclusion that poisonous and remedial agents exert a specific influence over particular tissues, by modifying the processes which normally take place in them, in virtue of a certain chemical relation subsisting between the agent on the one hand, and the tissue or some of its products on the other. The recent experiments of Mr. Blake on the action of poisons lead to analogous results; for he has given strong reason to believe that all poisons, save those which have an immediate and violent local operation (such as the mineral acids) are absorbed into the blood, and carried to some particular tissue on which they have a specific effect; the time which is required for the influence of oxalic acid on the heart, or strychnia on the spinal cord, being strictly proportional in different animals to the time required for the conveyance of other agents along the same course. Again, the very same doctrine, expressed almost in the words of Liebig, was propounded in a paper read some months since in the Medico-Chirurgical Society (to appear, we understand, in the forthcoming volume of the Transactions) by Dr. W. Budd; in which the author brings together a number of facts regarding the local action of remedies, and the *symmetrical* changes produced in the body by many diseases, in proof of the opinion, that all such local actions must be the result of the presence of the agents in the circulating blood, and of an "elective affinity" between each and the tissue or organ on which it operates. The views of Liebig render more precise our ideas of this local action; and take from Dr. Budd's supposition the uncertain character of an hypothesis, conferring upon it the definiteness of a theory.

VI. *Theory of Disease.* Our limits prevent us from adding more than a very few observations on the two remaining divisions of the work before us. We the less regret this, since it appears to us that our author's theory of disease is the least successful part of it. He attempts to reduce to one general expression that which we believe no real pathologist would regard as capable of such generalization; although the "unity of disease" may be a very specious dogma for a practitioner to trumpet forth, who wishes to attract public attention; and the curability of all maladies by one nostrum may well serve the purposes of the empiric. As the normal or physiological state of *health* depends on an immense variety of conditions, so an alteration in any one of these conditions will produce a pathological state—that of *disease;* and we do not see that we are much aided in our conception of it by any such definition of it as the following: "Disease occurs when the sum of vital force, which tends to neutralize all causes of disturbance (in other words, when the resistance offered by the vital force) is weaker than the acting cause of disturbance." In pursuing this idea into its details, it appears to us that our author's want of acquaintance with scientific pathology renders him inadequate to the object he has proposed; and we may especially advert to his explanation of a *febrile paroxysm,* as exhibiting an utter ignorance of the nature of that disturbance.

VII. The *Theory of Respiration,* with which the text concludes, appears to us to contain little novelty. The chief point on which the author dwells, is the agency of the globules in conveying oxygen from the lungs to the systemic capillaries, and in bringing back carbonic acid from these to be discharged at the lungs, an idea which has long been familiar to physiologists. He considers this to be the exclusive function of the globules, affirming that physiology has proved that the globules take no share in the function of nutrition; in which statement Dr. Barry would consider him as somewhat premature. He brings forward, however, an extremely interesting view of the function of the *iron,* which is well known to exist in the blood-corpuscles. If it be regarded as originally in the state of protoxide, it will be converted into peroxide by exposure to atmospheric air in the lungs; in the systemic capillaries it will give off half of its oxygen, and be reduced to the state of protoxide; this protoxide will combine with the carbonic acid set free in the same situation, and the iron will return to the lungs in the state of carbonate. In the lungs it will change its equivalent of carbonic acid for one of oxygen, which will be conveyed to the tissues as before. Now although the fibrin and albumen of venous blood undergo a change by exposure to air in the lungs, and undergo a converse change in the systemic capillaries, it cannot be questioned that the chief alteration takes place in the contents of the blood-corpuscles; and the idea of Liebig as to the nature of that change appears to us peculiarly satisfactory. It fully accounts for the phenomenon; since the result of analyses and calculations proves that the amount of iron present in the blood, if in a state of protoxide, is sufficient to furnish the means of carrying or transporting twice as much carbonic acid, as can possibly be formed by the oxygen absorbed in the lungs. It also explains other well-known facts, in regard to the asphyxiating influence of minute quantities of certain gases and vapours, such as sulphuretted hydrogen and prussic acid, of which no other account has been offered; since these agents have a powerful effect upon iron, when alkalies are present; and free alkali is never absent in the blood.

The latter part of the volume is occupied by a copious appendix, containing a vast amount of important analytical evidence, referred to in the body of the work.

In conclusion, we have only to add to the observations with which we commenced, our tribute of praise to the able translator, Dr. W. Gregory, for the admirable manner in which he has placed this most important treatise before the English reader; and our renewed recommendation to all who desire to share in the honour of themselves advancing science, or who merely wish the credit of keeping pace with it, to "read, mark, learn, and inwardly digest" its valuable contents.

---

